# Comprehensive Profiles of mRNAs and miRNAs Reveal Molecular Characteristics of Multiple Organ Physiologies and Development in Pigs

**DOI:** 10.3389/fgene.2019.00756

**Published:** 2019-08-28

**Authors:** Muya Chen, Yi Long Yao, Yalan Yang, Min Zhu, Yijie Tang, Siyuan Liu, Kui Li, Zhonglin Tang

**Affiliations:** ^1^Research Centre for Animal Genome, Agricultural Genome Institute at Shenzhen, Chinese Academy of Agricultural Sciences, Shenzhen, China; ^2^Genome Analysis Laboratory of the Ministry of Agriculture, Agricultural Genome Institute at Shenzhen, Chinese Academy of Agricultural Sciences, Shenzhen, China; ^3^Institute of Animal Science, Chinese Academy of Agricultural Sciences, Beijing, China

**Keywords:** mRNAs, miRNAs, multiple organ, skeletal muscle, pig

## Abstract

The pig (*Sus scrofa*) is not only an important livestock animal but also widely used as a biomedical model. However, the understanding of the molecular characteristics of organs and of the developmental skeletal muscle of the pig is severely limited. Here, we performed a comprehensive transcriptome profiling of mRNAs and miRNAs across nine tissues and three skeletal muscle developmental stages in the Guizhou miniature pig. The reproductive organs (ovary and testis) had greater transcriptome complexity and activity than other tissues, and the highest transcriptome similarity was between skeletal muscle and heart (*R* = 0.79). We identified 1,819 mRNAs and 96 miRNAs to be tissue-specific in nine organs. Testis had the largest number of tissue-specific mRNAs (992) and miRNAs (40). Only 15 genes and two miRNAs were specifically expressed in skeletal muscle and fat, respectively. During postnatal skeletal muscle development, the mRNAs associated with focal adhesion, Notch signaling, protein digestion, and absorption pathways were up-regulated from D0 to D30 and then down-regulated from D30 and D240, while genes with opposing expression patterns were significantly enriched in the oxidative phosphorylation and proteasome pathways. The miRNAs mainly regulated genes associated with insulin, Wnt, fatty acid biosynthesis, Notch, MAPK, TGF-beta, insulin secretion, ECM–receptor interaction, focal adhesion, and calcium signaling pathways. We also identified 37 new miRNA–mRNA interaction pairs involved in skeletal muscle development. Overall, our data not only provide a rich resource for understanding pig organ physiology and development but also aid the study of the molecular functions of mRNA and miRNA in mammals.

## Introduction

Intensive transcriptome sequencing is increasingly being used to study the mechanisms of organ physiology and development, with a goal of understanding the genetic expressions of tissue-specific diseases (Lage et al., 2008; Koh et al., 2014). Since the expressions, and thus the functions, of many genes vary between tissue types, the study of tissue-specific genetic expression describes those transcriptional variations and provides insights into the underlying genetic mechanisms in each specific tissue type. Thus, obviously the construction of comprehensive tissue-specific transcriptome profiles for both humans and model organisms is of great importance. Indeed, tissue-specific expression of both protein-coding and non-coding RNAs in both humans and mice has been well studied (Roux et al., 2012; Szabo et al., 2015; Zeng et al., 2016; Iwakiri et al., 2017) and the results have been used to elucidate organ physiologies and diseases. For example, the identification of tissue-specific genes was used to build a tissue-specific gene database for human cancers (Kim et al., 2018). However, while many studies have accumulated both mouse and human tissue-specific transcriptome data (Zhang et al., 2015a), RNA-seq transcriptome analyses across tissues and developmental stages of other mammals are still relatively scarce.

Wild *Sus scrofa* (pig) was domesticated approximately 9,000 years ago and has become one of humankind’s most important livestock animals (Giuffra et al., 2000). Because of its similarity with humans in body size, lifespan, anatomy, and other distinct physiological characteristics, the pig has become a model in many disciplines of biomedical research, such as pharmacology, obesity, oncology, cardiology, and many more (Groenen et al., 2012). While pig mRNA and miRNA profiles have been obtained for several tissues, such as adipose, liver, skeletal muscle (Hou et al., 2012; Tang et al., 2015), and testis (Zhang et al., 2015b), those analyses focused mainly on a single organ or developmental stage. Systematic studies of miRNA and mRNA spatiotemporal expression patterns and their interactions in multiple organs and developmental stages are still needed to further understand their physiological functions and development, which would aid both biomedical research and pig husbandry. Interactions between miRNAs and their target mRNAs play important roles in regulating various biological processes (Bai et al., 2015), so identification of those interactions provides insights into the different mechanisms at work in each tissue type (Neville et al., 2011; Wang et al., 2012b).

In this study, we used high-throughput transcriptome sequencing to comprehensively explore *S. scrofa* mRNA and miRNA profiles in nine different tissues and three developmental stages of skeletal muscles. First, we systematically analyzed expression characteristics of protein coding genes (PCGs) and miRNAs in nine tissues, identifying the tissue-specific and -associated PCGs/miRNAs and then exploring the interactions of miRNAs with their target mRNAs in nine organs. Finally, we detected a set of miRNA–mRNA interaction pairs potentially associated with postnatal skeletal muscle development. Overall, this study provides a comprehensive profile of mRNAs and miRNAs in multiple organs and developmental stages in the pig and provides meaningful insights into tissue-specific metabolic regulation at the RNA level.

## Materials and Methods

### Animals and Organ Collection

In this study, we collected nine tissues (fat, heart, kidney, liver, lung, skeletal muscle, ovary, spleen, and testis) from the Guizhou miniature pig, one of the most primitive pig breeds in China and a source of high quality meat, at 240 days of age and two additional skeletal muscle samples at postnatal 0 and 30 days. These samples were collected from three biological individuals at each postnatal date (0, 30, and 240 days). All samples were rapidly isolated and immediately frozen in liquid nitrogen. All animal procedures were performed according to protocols approved by the Biological Studies Animal Care and Use Committee in Beijing Province, China.

### Isolation of Total RNA and Construction of RNA-seq Libraries

We extracted total RNA from various tissues at least three times, mixing RNA samples from each tissue type into one group per type, and small RNA library for each tissue group was produced. Polyacrylamide electrophoresis gel was used to purify the fragments of 18–30 nt, and then these fragments were ligated to adaptors on both 5′ and 3′ ends. After reverse-transcription amplification, the PCR products in length of 90-bp were isolated from 4 % agarose gels, and then sequenced on the Illumina HiSeq 2500 platform. The RNA-seq data for mRNA were deposited in the Gene Expression Omnibus (accession codes GSE73763) as our previous reports (Tang et al., 2017; Liang et al., 2017), and the reliability of transcriptome data has was verified by qRT-PCR in the study by Tang et al. (2017).

### RNA-seq Data Analysis

First, using custom scripts, we trimmed adapters from all RNA sequencing data and then mapped the processed reads from each sample to the *S. scrofa* reference genome (v10.2) using TopHat2 (Kim et al., 2013) (v2.0.12) with fr-frststrand and the following parameters: mate-inner-dist 20, mate-std-dev 50, microexon-search segment-length 25, and segment-mismatches 2. Alignment results from each sample were then processed using Cufflinks (v2.2.1) with known annotations for transcript assembly (fr-firststrand and min-frags-per-transfrag 3) and then the consensus transcriptome was merged. Finally, we used HTseq-count (v0.6.1) required strand-specific counting (Anders et al., 2015) to quantify genes and transcripts. We then calculated the reads per kilobase million (RPKM), counted on read pairs in cases of paired ends, for the PCGs.

### miRNA-seq Data Analysis

Raw sequencing reads were obtained after removing reads without a 3′ primer or insert tag, reads with polyA or 5′ primer contaminants, and reads shorter than 18 nt or longer than 32 bp. Then, the clean reads that could be annotated and aligned to rRNAs, snoRNAs, and tRNAs in the Rfam database (http://rfam.xfam.org) (Nawrocki et al., 2015) were discarded. We mapped the remaining reads to the *S. scrofa* reference genome (v10.2) using miRDeep2(v2.0.0.8) software (Friedlander et al., 2012). The sequences of known mature miRNAs and their precursors were downloaded from miRBase (http://www.mirbase.org) (Kozomara and Griffiths-Jones, 2014), and the expression level of each miRNA was normalized using the transcripts per kilobase million (TPM) method.

### Gene Expression Analyses

In our analyses, all PCG-miRNAs that had an RPKM or TPM greater than 0.1 in at least one sample were considered to be expressed. In addition, Pearson correlation coefficients were calculated to examine the similarities and correlations of mRNA expression in different samples. In our analyses, universally expressed genes are tissue-conserved expressed genes whose RPKM values are greater than 10 in every tissue. For miRNAs, two criteria were used to define universally expressed miRNAs across different tissues: 1) the TPM value in every tissue was more than 1, and 2) the coefficient of variation across all tissues was less than 0.5.

The tissue-associated genes for any given tissue were identified according to a previous study with the *Z*-score cutoff ≥ 1.5 and RPKM ≥ 1 (Li et al., 2014). We identified tissue-specific genes as having RPKM or TPM ≥ 10, with expression levels in a given tissue being greater than 10-fold higher than the mean expression value of any other tissues.

### Co-Expression Network Analysis

We used RNA libraries of nine different tissue types, with all samples collected at postnatal day 240, for network construction. Based on the mRNA expression matrix, we constructed a weighted co-expression network using BioLayout Express (3D) with a Pearson correlation threshold cutoff ≥ 0.90 and a Markov clustering algorithm of 2.2.

### miRNA–mRNA Interaction Analyses

miRNA–host gene co-expression pairs were identified based on the following pipeline: 1) the miRNA coordinate overlapped 100% of the protein-coding gene; 2) the host gene and the miRNA are transcribed from the same strand of DNA; 3) the miRNA does not have extra copies in other parts of the genome, since the transcription of each copy of the miRNA gene could be regulated by different mechanisms that would confound the results of our analyses; 4) the intragenic miRNAs and the host genes must be expressed in at least five tissues (TPM or RPKM ≥ 0.1); and 5) significant correlations were identified as *p* < 0.05 and *r* > 0.6 (Kaminski et al., 2013; Lin et al., 2016).

mRNA and miRNA pairs were subjected to Pearson correlation analysis and those pairs with *r* < −0.5 were chosen for further investigation. Then, we used the RNAhybrid (v2.1.2) (Kruger and Rehmsmeier, 2006) and TargetScan algorithms (Riffo-Campos et al., 2016) to detect whether the 3′-untranslated region (3′UTR) of the mRNA in each pair matched the seed region of the corresponding miRNA. The pairs that satisfied those two conditions were used in both KEGG pathway analysis and Gene Ontology (GO) enrichment analysis to further investigate the biological processes and functions associated with those negative correlations. These analyses were based on human annotation using the Database for Annotation, Visualization, and Integrated Discovery (DAVID) web server (http://david.abcc.ncifcrf.gov/) with the EASE value set to 0.05 (Huang et al., 2007; Huang Da et al., 2009).

### Differential Expression Analysis

We used R (v3.2.0) DESeq2 package (Love et al., 2014) for differential expression analyses of PCGs and miRNAs in skeletal muscle at three developmental (D) stages (D0, D30, and D240 postnatal days). Analysis was performed between muscle_D0 and muscle_D30 and between muscle_D30 and muscle_D240. In this study, significant differentially expressed genes (DEGs) met the criteria log2-FC ≥ 1 and FDR < 0.05, where FC is fold change and FDR is false discovery rate, and those acceptable DEGs were further examined with GO and KEGG analyses, as above.

### Vector Construction, Cell Culture, and Dual Luciferase Reporter Assay

Two miRNA–mRNA interaction pairs (*ACTN4*/ssc-miR-133a-3p *and Prox1*/ssc-miR-338) were randomly selected to verify the expression profiles of miRNA and its target mRNA. Through the PCR method, the 3′UTR fragments (3′UTR-wt) flanking miRNA binding sites of these two genes were amplified and then cloned into pmirGLO Dual-Luciferase Vector through the Homologous Recombination Kit (Qingke, China). The mutant types of these two genes with the 3′UTR region (3′UTR-edt) were made by the Homologous Recombination Kit (Qingke, China) and confirmed by sequencing. Primer sequences are listed in **Table 1**.

**Table 1:** Primer sequences.

Name	Primer sequences (5′–3′)	Application
ACTN4 3′UTR	F: TCTAGTTGTTTAAACGAGCTCGCCTCTTGCTCCCGTAAT R: CAGGTCGACTCTAGACTCGAGGGAGCAAAACCATCCACTA	Amplification and vector construct
PROX1 3′UTR	F: TCTAGTTGTTTAAACGAGCTCGTAGTCGCAGTCCCCTTT R: CAGGTCGACTCTAGACTCGAGTAAACTAAAGGCGGAAGG	Amplification and vector construct
ACTN4 3′UTR MT	F: TCTAGTTGTTTAAACGAGCTCTATGTTCTGAAATCGTAGTT R: CAGGTCGACTCTAGACTCGAGCCCTCCCTTGCGAACAC	Mutation vector construct
PROX1 3′UTR MT	F: TCTAGTTGTTTAAACGAGCTCTGGGTCTCTGAAAGTT R: CAGGTCGACTCTAGACTCGAGCCCCAGAAGGGTGGTTTATC	Mutation vector construct

The HEK293 cells were cultured at 37°C with Dulbecco’s modified Eagle’s medium (Sigma), 10% FBS (Gibco), 1% penicillin/streptomycin (Gibco), and 5% CO_2_. The miR-and -133b mimics (double-stranded RNA oligonucleotides) and negative control duplexes were synthesized by GenePharma. The pmirGLO-3′UTR-wt, pmirGLO-3′UTR-mt and miRNA (mimic/negative control) were co-transfected into HEK293 cells. The co-transfection assays were performed in 12-well plates with Lipofectamine 2000 reagent (Invitrogen) according to the manufacturer’s instructions and harvested after 24 h. Finally, the dual-luciferase assay system (Promega) was used to examine the activity of renilla and firefly luciferase.

## Results

### Overview of mRNA and miRNA Profiling

**Figure 1:**
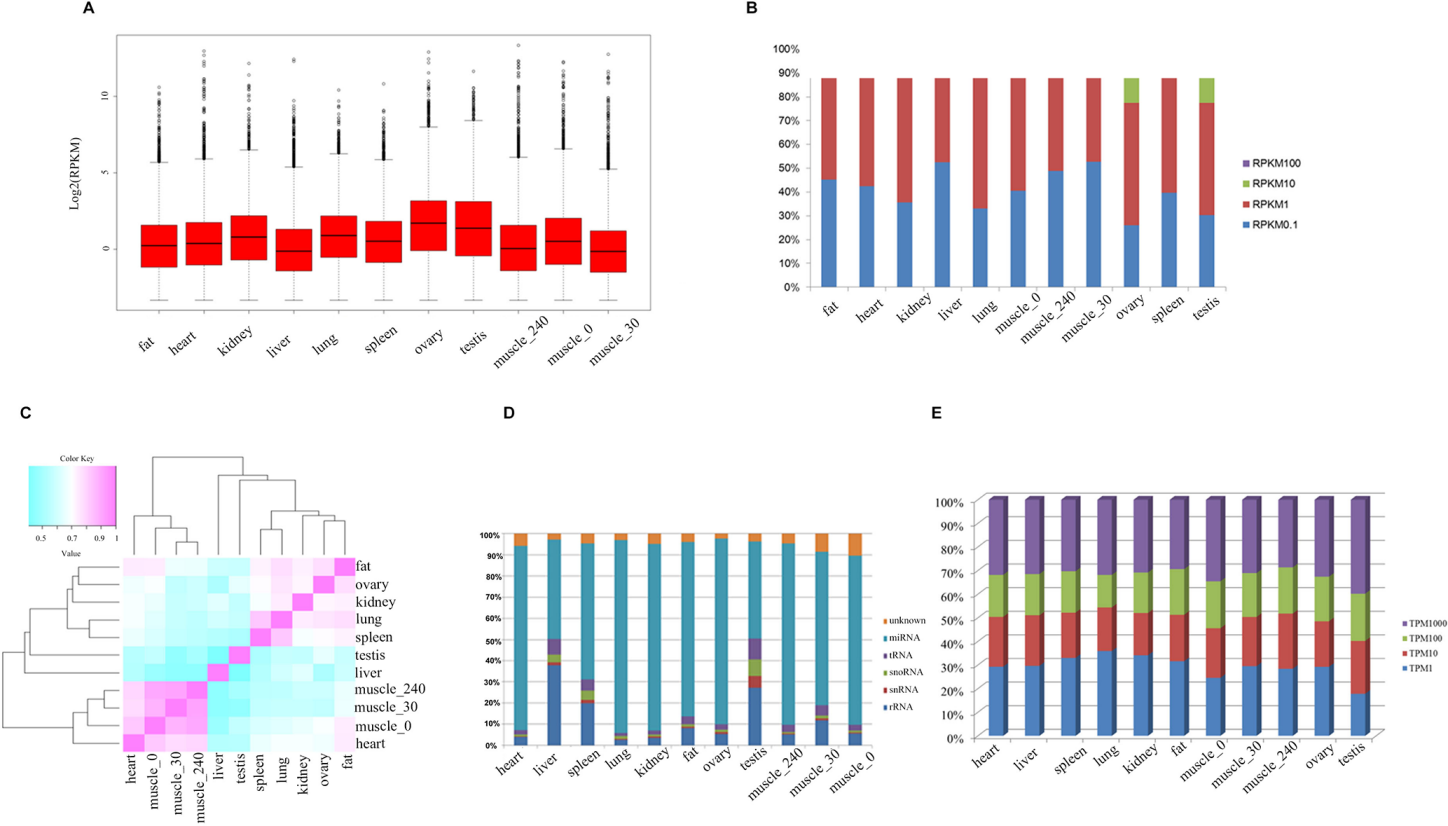
The transcriptome profile of multiple tissues in pigs. **(A)** Genome-wide mean expression value profile. **(B)** Genome-wide RPKM distribution of mRNA. **(C)** Hierarchical clustering generated using Pearson correlation coefficients of log2-transformed RPKM (mRNA) values. **(D)** Composition of the RNA library in each tissue. **(E)** Genome-wide RPKM distribution of miRNA.

To systematically investigate genome-wide expression profiles of *S. scrofa* PCGs and miRNAs, we first performed RNA-seq and small RNA-seq on nine tissues, as well as on skeletal muscle tissue from three developmental stages of Guizhou miniature pigs. A total of 824,887,548 reads were obtained for RNA-seq analysis, as we described in our previous study (Liang et al., 2017). We then measured the expression abundance of PCGs in each tissue (**Figure 1A**), detecting 18,576 expressed PCGs (RPKM > 0.1) representing 85.97% of annotated PCGs in the porcine reference genome, and 46.14% of these PCGs were constitutively expressed through all selected tissues. The numbers of expressed PCGs ranged from 10,845 to 15,552 in the nine different tissues (**Table 2**). The smallest numbers of expressed PCGs were in skeletal muscle and the largest numbers were in the reproductive tissues (ovary and testis). Also, the distribution of genes with high RPKM (>10) values was larger in the reproductive tissues than in the other tissues (**Figure 1B**). These findings indicated that transcriptome complexity and activity in the reproductive system were higher than those in other tissues. We next examined the similarities and correlations between tissues based on global expression profiling. Clustering analysis suggested that the highest transcriptome similarity was shown between skeletal muscle and heart (Pearson correlation, *R* = 0.79), whereas the testis and liver showed the lowest expression correlation (Pearson correlation, *R* = 0.41) (**Figure 1C**).

**Table 2:** Numbers of expressed genes in different tissues.

	RPKM1	RPKM10	RPKM100	RPKM1000	Total
Fat	6,015	6,367	843	56	13,281
Heart	5,278	6,032	1,008	89	12,407
Kidney	5,023	7,541	1,436	73	14,073
Liver	6,282	4,867	763	77	11,989
Lung	4,988	8,559	1,474	54	15,075
Muscle_0	4,766	5,746	1,151	124	11,787
Muscle_240	5,297	4,627	820	101	10,845
Muscle_30	5,976	4,752	574	62	11,364
Ovary	3,928	7,792	3,171	260	15,151
Spleen	5,193	6,928	936	55	13,112
Testis	4,722	7,339	3,277	214	15,552

Small RNA analysis revealed 131,789,681 high-quality clean reads, accounting for 93.64% of the total reads. Analysis of the size distribution of all reads showed that the major class of reads peaked at 22–23 nt within most of the libraries. However, in liver and testis, the majority of clean reads were at 28–30 nt, followed by 22 nt, thus implying that Piwi-interacting RNAs were enriched in those two tissues. For all 11 libraries, 99,812,523 (75.75%) of the clean reads were mapped to the porcine reference genome. The composition of each RNA library (**Figure 1D**) shows our findings that 319 known and 442 novel miRNAs (TPM > 0.1) were expressed in all the tissues we investigated. The number of expressed miRNAs in these libraries ranged from 475 to 594, including 206 to 292 novel miRNAs and 269 to 302 known miRNAs, respectively (**Table 3**). The miRNAs with high RPKM (>10) values had a larger distribution in testis than in other tissues (**Figure 1E**).

**Table 3:** Numbers of miRNA identified in different tissues.

Tissue	Novel	Known	Total
Heart	229	283	512
Liver	260	283	543
Spleen	292	302	594
Lung	275	293	568
Kidney	258	290	548
Fat	286	299	585
Muscle_0	216	293	509
Muscle_30	282	294	576
Muscle_240	206	269	475
Ovarium	248	292	540
Testis	222	283	505
Total	439	319	758

### Universally and Specifically Expressed mRNAs and miRNAs Across Tissues

Focusing on universally expressed mRNAs and miRNAs, we found that 209 mRNAs (**Table S1**), representing tissue-conserved expressed genes whose RPKM values were greater than 10 in all tissues, were abundantly and stably expressed. This dataset included some well-known housekeeping genes such as *GAPDH*, *ACTB*, *RPS18*, *B2M*, *RPL4*, *RPL37*, and *RPL38*. According to GO analysis, these genes were significantly enriched in the areas of translation, peptide biosynthetic, and amide biosynthetic (**Table S2**), indicating that they have important roles in maintaining essential basal cellular functions. Additionally, we identified 43 universally expressed miRNAs (**Table S3**). One of those miRNAs, miR-16, is most likely an important biomarker for several diseases including lung cancer, rheumatoid arthritis, and sepsis (Wang et al., 2012a; Sromek et al., 2017; Dunaeva et al., 2018) in humans; is abundantly expressed in all tissues; and has been used as a control in several systems, including animal models. These miRNAs may serve as candidate reference to normalize miRNA expression across tissues.

In order to capture the functional differences in gene expression between tissues, we next analyzed tissue-specific mRNA and miRNA. As in a previous study (Li et al., 2017), the genes whose abundance in one tissue was more than fourfold the mean expression value of that in other tissues were defined as tissue-specific genes. While for some genes the expression levels were very low, we defined the tissue-specific mRNAs and miRNAs as the genes whose abundance in one tissue was more than 10-fold the mean expression value in other tissues, with RPKM or TPM ≥10. Finally, we identified 1,819 tissue-specific PCGs, with the number ranging from 15 to 992 for a given tissue (**Table S4**). Testis had the largest number of tissue-specific genes (54.5%, 992/1819), compared with other tissues. In contrast, only 15 genes were specifically expressed in skeletal muscle. We found that the expression of *GAPDH* was significantly higher in skeletal muscle than in other tissues, a result consistent with a previous mouse study (Fortes et al., 2016). Meanwhile, we identified 96 tissue-specific miRNAs (including 48 novel and 48 known miRNAs). The number of miRNAs ranged from 2 to 40 for a given tissue. The largest numbers of tissue-specific miRNAs were found in testis and only two tissue-specific miRNAs were found in fat (**Table 4**). The well-known myomiRs (miRNA-1, miR-133a/b, and miR-206) were specifically expressed in skeletal muscle. In testis, the top two tissue-specific miRNAs, miR-34c and miR-202-5p, were shown to possess an important influence in spermatogenesis regulation (Dabaja et al., 2015; Wang et al., 2018b). For fat, only one known miRNA (miR-224), which was reported to have an important role in adipogenesis development, was specifically expressed (Peng et al., 2013).

**Table 4:** Tissue-specific miRNA in different tissues.

Tissue	Known miRNAs	Novel miRNAs
Heart	ssc-miR-208b, ssc-miR-490, ssc-miR-499-3p, ssc-miR-499-5p, ssc-miR-7136-5p	novel_14_8571, novel_7_25708
Liver	ssc-miR-122, ssc-miR-192, ssc-miR-194b-5p, ssc-miR-365-3p, ssc-miR-885-3p, ssc-miR-885-5p	novel_13_6504, novel_14_8741, novel_9_28064
Spleen	ssc-miR-142-5p, ssc-miR-145-5p, ssc-miR-150, ssc-miR-20b, ssc-miR-342, ssc-miR-7138-5p	novel_10_3375, novel_2_14974, novel_GL896302.1_32316
Lung	ssc-miR-138,ssc-miR-205,ssc-miR-92b-3p	novel_5_19972, novel_5_19974
Kidney	ssc-miR-10a-3p, ssc-miR-10a-5p, ssc-miR-196b-5p, ssc-miR-204, ssc-miR-429	novel_1_778, novel_6_20939
Fat	ssc-miR-224	novel_5_18915
Skeletal muscle	ssc-miR-1, ssc-miR-133a-3p, ssc-miR-133a-5p, ssc-miR-133b, ssc-miR-206	novel_17_12538, novel_2_14142, novel_2_15377, novel_3_17211, novel_X_29629
Ovarium	ssc-miR-132, ssc-miR-135, ssc-miR-212, ssc-miR-7857-3p	novel_8_26925, novel_9_28118
Testis	ssc-miR-137, ssc-miR-153, ssc-miR-182, ssc-miR-202-3p, ssc-miR-202-5p, ssc-miR-216, ssc-miR-217, ssc-miR-221-3p, ssc-miR-222, ssc-miR-34c, ssc-miR-708-3p, ssc-miR-708-5p	novel_11_3521, novel_13_5908, novel_14_7685, novel_15_10051, novel_16_11835, novel_16_11837, novel_2_14220, novel_2_14976, novel_3_16101, novel_4_17487, novel_5_18832, novel_5_20192, novel_5_20217, novel_5_20223, novel_9_28635, novel_9_28958, novel_9_29013, novel_GL893854.2_31046, novel_X_29642, novel_X_29643, novel_X_30198, novel_X_30204, novel_X_30206, novel_X_30208, novel_X_30210, novel_X_30214, novel_X_30215, novel_X_30218

### Tissue-Associated mRNAs Capture the Structure and Functional Features of Different Tissues

**Figure 2:**
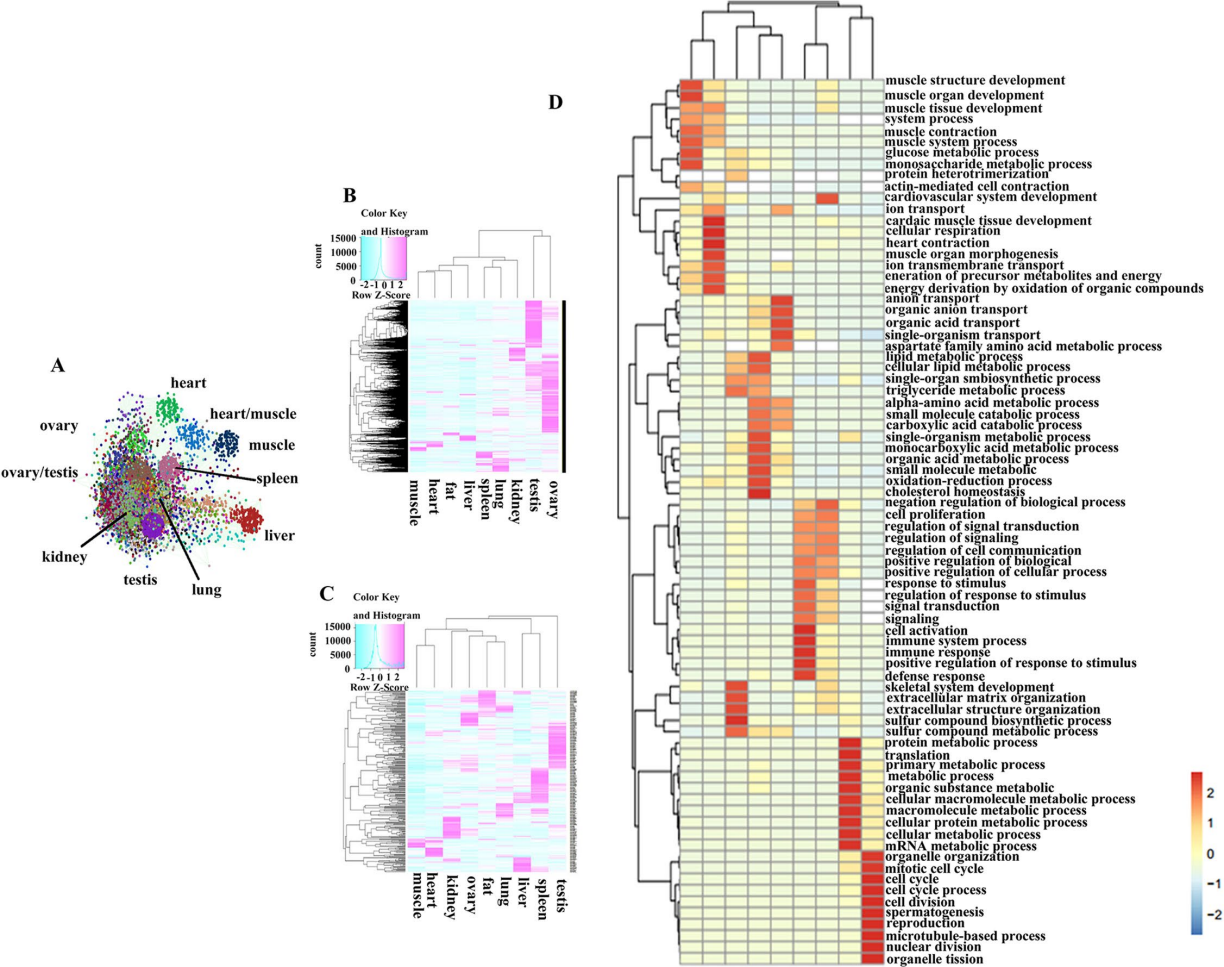
The expression characteristic of tissue-associate genes. **(A)** Co-expression network of the protein coding genes. **(B)** Heatmap of tissue-associated mRNA in different pig tissues. **(C)** Heatmap of tissue-associated miRNA in different pig tissues. **(D)** GO biological process analysis of tissue-associated mRNA.

A weighted and undirected co-expression network analysis was performed to understand interactions between PCGs, and it generated 229 distinct clusters containing 13,894 nodes (**Figure 2A**). Cluster names were based on the tissues in which the genes were expressed the most. The three largest clusters (including 4,997 nodes) were groups of highly expressed genes in ovary and testis. These results indicated that the majority of PCGs showed a tissue-restricted expression pattern. Thus, we tried to capture the basic characteristics of gene expression for each tissue by using ‘‘tissue-associated genes,” genes that were highly expressed in one tissue relative to other tissues (see *Materials and Methods*). The number of associated PCGs and miRNAs (**Tables S5**, **S6**) for a given tissue ranged from 287 to 5,606 and from 28 to 132, respectively, suggesting that the tissues with higher transcriptional activities, such as testis, had more associated genes (**Figures 2B, C**). GO analysis, based on tissue-associated PCGs, revealed physiological features for each organ (**Figure 2D**). For example, GO terms for muscle tissue development were significantly enriched in skeletal muscle and heart, while the genes associated with spermatogenesis, lipid metabolism, and immune response were enriched in testis, adipose, and spleen, respectively. Also, we observed common GO terms shared in different tissues. For instance, the GO term related to the cell cycle and metabolic processes was obviously enriched in both ovary and testis, confirming that the reproductive tissues were highly proliferative. The GO terms for small-molecule catabolic and other metabolic processes were markedly enriched in liver and kidney. The genes associated with stimulus response and signal transduction were significantly enriched in lung and spleen. In summary, GO terms of tissue-associated genes agreed with the physiologies of the corresponding organs (**Table S7**).

### Differentially Expressed mRNA and miRNA During Skeletal Muscle Development

**Figure 3:**
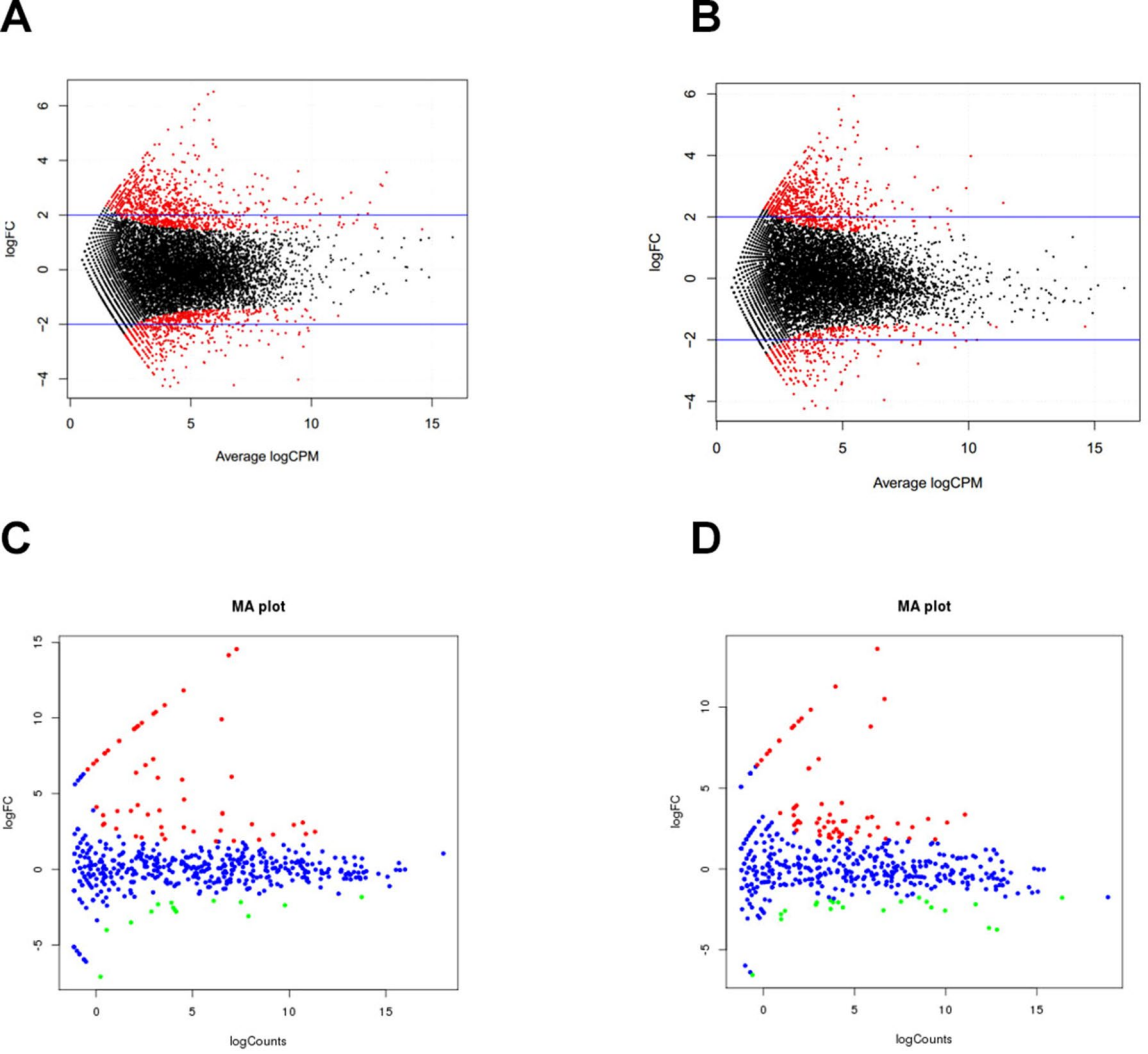
Differentially expressed genes during skeletal muscle development. **(A)** D0 vs. D30 mRNA. **(B)** D30 vs. D240 mRNA. **(C)** D0 vs. D30 miRNA. **(D)** D30 vs. D240 miRNA.

**Figure 4:**
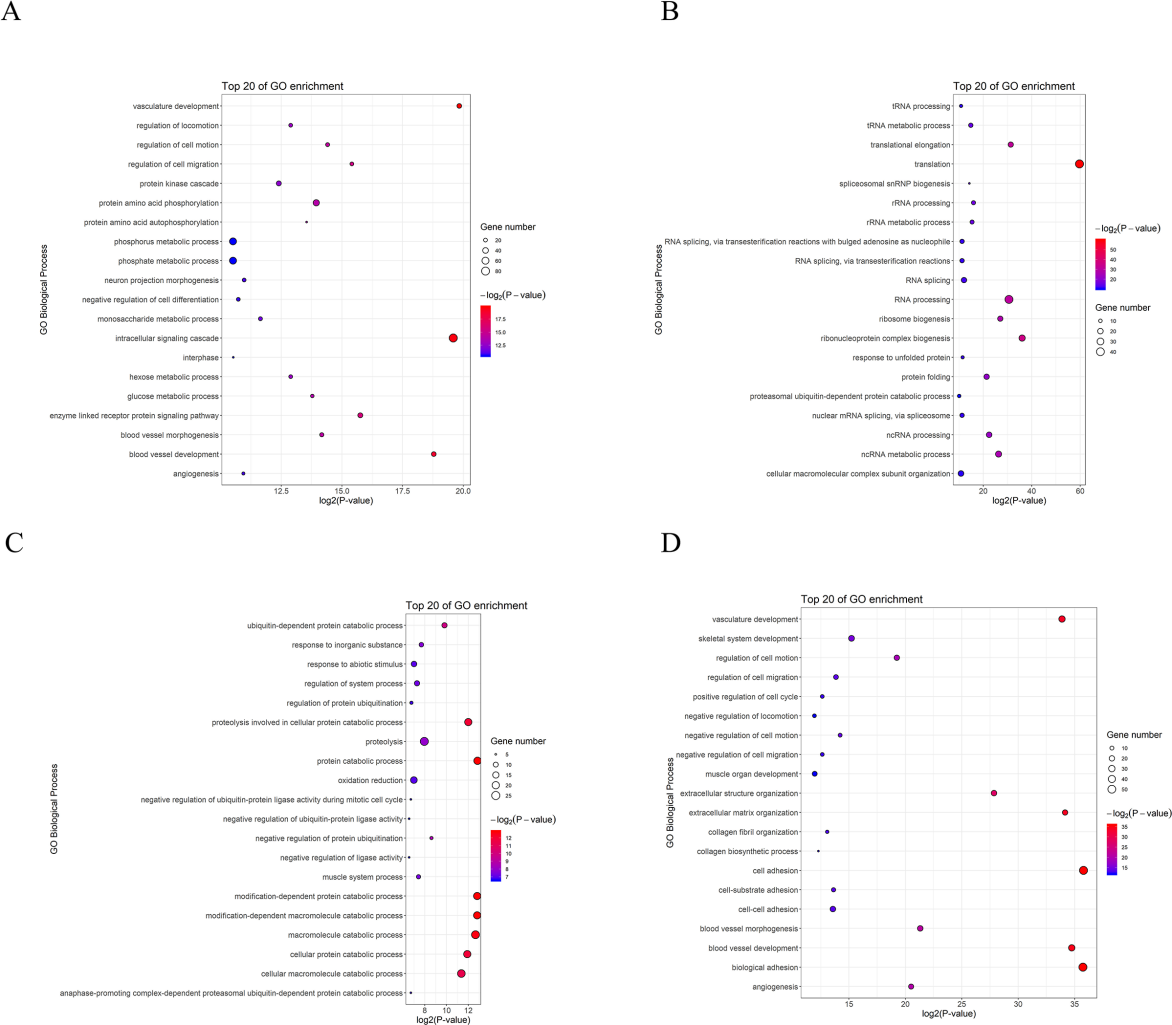
GO analysis of differentially expressed genes during skeletal muscle development. **(A)** D0 vs. D30 up-regulated genes. **(B)** D0 vs. D30 down-regulated genes. **(C)** D30 vs. D240 up-regulated genes. **(D)** D30 vs. D240 down-regulated genes.

To understand postnatal skeletal muscle development, we assessed the differentially expressed PCGs and miRNAs in skeletal muscle across three developmental stages at 0, 30, and 240 days after birth (D0, D30, and D240, respectively). Between D0 and D30, we detected 1,515 DEGs (**Table S8**), including 911 up-regulated and 604 down-regulated genes, respectively (**Figure 3A**). GO analysis (**Table S9**) suggested that the up-regulated genes were involved mainly in vasculature development, the intracellular signaling cascade, blood vessel development, and enzyme linked receptor protein signaling pathway (**Figure 4A**), and the down-regulated genes were associated mainly with translation, ribonucleoprotein complex biogenesis, and RNA and ncRNA processing (**Figure 4B**). Between D30 and D240, we identified 1,011 DEGs (**Table S10**) including 338 up-regulated and 673 down-regulated genes (**Figure 3B**). According to GO analysis (**Table S9**), up-regulated genes were involved mainly in protein catabolic process, modification-dependent macromolecules, and modification-dependent protein catabolic process (**Figure 4C**). The down-regulated genes were involved mainly in cell adhesion, biological adhesion, and skeletal systems development (**Figure 4D**). These findings indicated that proliferative cell activity decreased, while cellular metabolic ability increased with age during postnatal skeletal muscle development and growth.

**Figure 5:**
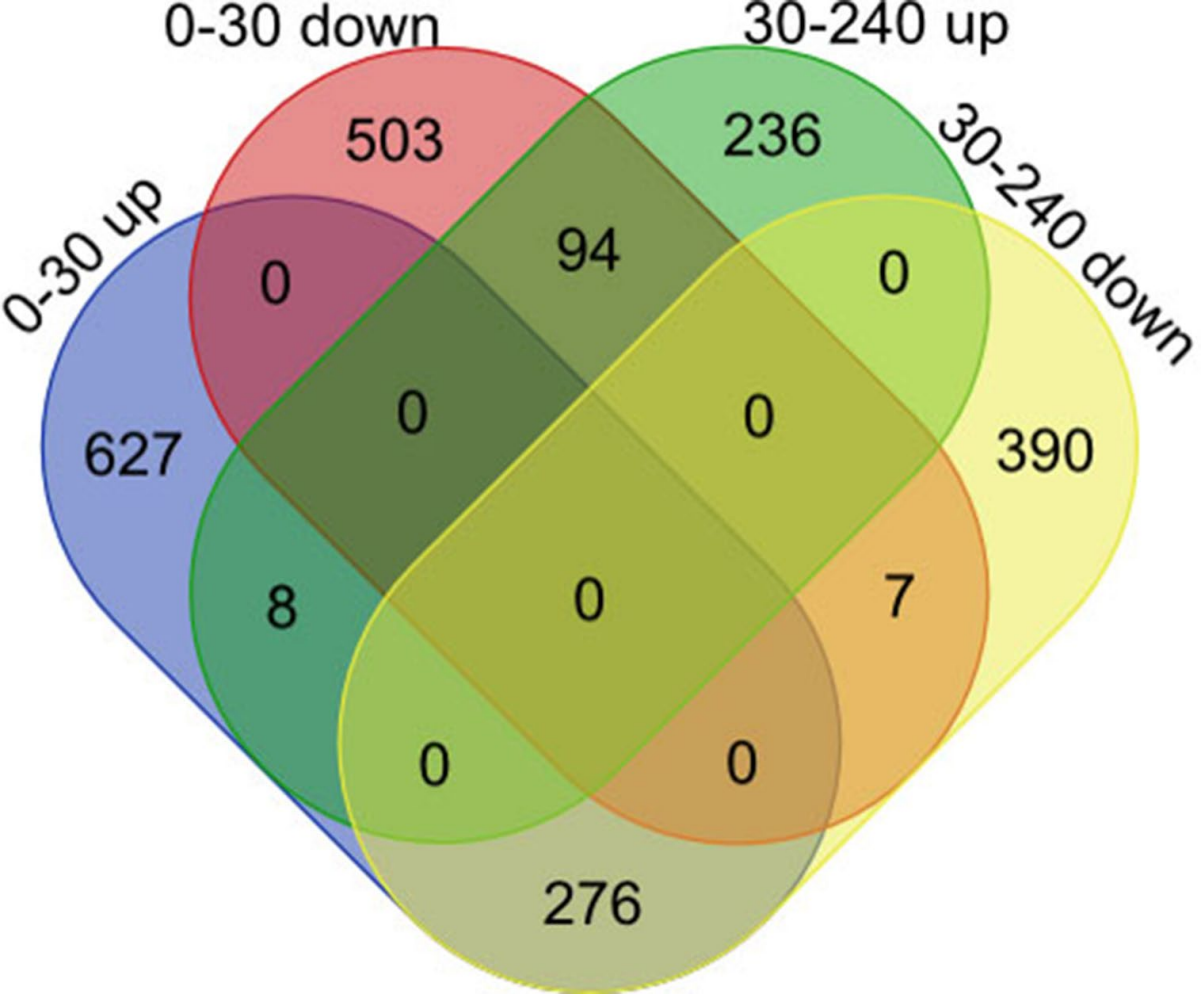
Co-expression pattern analysis for genes during skeletal muscle development.

Subsequently, we focused on a series of dynamic expression patterns exhibited during skeletal muscle development. Venn diagram (**Figure 5**) analysis for DEGs demonstrated that the greatest overlap (276 genes) occurred between both up-regulated genes in group D0 versus D30 and down-regulated genes in group D30 versus D240 (**Table S11**). The largest cluster of overlapping genes were significantly associated with vasculature, the cardiovascular and circulatory systems, and blood vessel development, and obviously gathered in the focal adhesion, Notch signaling, and protein digestion and absorption pathways (**Tables S12**, **S13**). The second largest overlap cluster contained 94 genes and were present between the down-regulated genes in group D0 versus D30 and the up-regulated genes in group D30 versus D240 (**Table S11**). These overlapping genes functioned in the ATP metabolic process, purine ribonucleoside triphosphate metabolic process, and ribonucleoside triphosphate metabolic process and were significantly enriched in KEGG pathways for oxidative phosphorylation, proteasome, and Parkinson’s disease (**Tables S12**, **S13**). However, there were only eight genes up-regulated and seven genes down-regulated throughout D0 to D240 (**Table S11**).

These miRNAs also played an important role in skeletal muscle development. In this study, we identified 70 and 85 differentially expressed miRNAs in groups D0 versus D30 and D30 versus D240, respectively (**Tables S14**, **S15**). Between D0 and D30, 56 miRNAs were up-regulated and 14 were down-regulated (**Figure 3C**). Functional analysis suggested that the genes targeted by down-regulated miRNAs were significantly enriched in the insulin, Wnt, and Notch signaling pathways and in fatty acid biosynthesis, while the target genes for up-regulated miRNAs associated mainly with the MAPK and TGF-beta signaling, insulin secretion, and ECM–receptor interaction pathways. In groups D30 versus D240, we detected 21 up-regulated and 64 down-regulated miRNAs, respectively (**Figure 3D**). The targets for the up-regulated miRNAs were significantly involved in the MAPK, focal adhesion, and insulin signaling pathways, while the targets for the down-regulated miRNAs were enriched mainly in the MAPK, TGF-beta, Notch, calcium, Wnt, insulin secretion, and focal adhesion pathways.

### miRNA–mRNA Interaction Network Associated With Skeletal Muscle Development

miRNAs can affect gene expression by inhibiting protein translation or by causing mRNA degradation (Tang et al., 2015). When we evaluated the expression relationships between miRNA and mRNA using Pearson correlations, we detected 253,057 miRNA–mRNA interactions that were negatively correlated (*r* < −0.5) and 2,194 pairs (1,605 mRNAs and 263 miRNAs) with binding sites for miRNAs at mRNA 3′UTRs. GO enrichment analysis suggested that these miRNA–mRNA interactions associated mainly with protein catabolic processes, muscle cell differentiation, and muscle organ development. KEGG analysis revealed that the interactions were significantly enriched in pathways for the citrate cycle, axon guidance, purine and pyruvate metabolisms, as well as the gonadotropin-releasing hormone, MAPK, adipocytokine, and insulin signaling pathways. In addition, there were 79 miRNA–mRNA interaction pairs that demonstrated functions in muscle cell differentiation and muscle organ development (**Table S16**). Of them, 37 miRNA–mRNA pairs showed significant negative expression correlations (*r* < −0.5) through all three skeletal muscle development stages (**Table 5**). Many mRNA genes have been reported as regulators of either muscle differentiation or development, including *MyoD1* (Blum et al., 2012), *CSRP2* (Herrmann et al., 2006), *MBNL1* (Chen et al., 2016), *FHOD1* (Staus et al., 2011), *MET* (Park et al., 2015), *SOD1* (Sakellariou et al., 2018), *RCAN1* (Emrani et al., 2015), *SGCA* (Fougerousse et al., 1998), *SRF* (Ding et al., 2017), *MEF2A* (Yuan et al., 2014), *MTM1*(Bachmann et al., 2017), *LBX1* (Chao et al., 2011), *MEF2D* (Runfola et al., 2015), *IGFBP5* (Zhang et al., 2017), *PDGFA* (Tallquist et al., 2000), *AMOT* (Wang et al., 2018a), *PDPK1* (Mora et al., 2003), *SIRT2* (Arora and Dey, 2014), *SIX4* (Chakroun et al., 2015), *NOS1* (Villmow et al., 2015), *MYL1* (Burguiere et al., 2011), *ACTA1* (Hu et al., 2015), *PROX1* (Kivela et al., 2016), and *QKI* (Wu et al., 2017). These interaction pairs included 7 known and 27 novel miRNAs, of which the miRNAs ssc-miR-744 (Yang et al., 2015), ssc-miR-497 (Sato et al., 2014), ssc-miR-338 (Mcdaneld et al., 2009), ssc-miR-423-3p (Siengdee et al., 2015), and ssc-miR-133a-3p (Wang et al., 2018c) were reported to have important roles in myogenesis.

**Table 5:** The expression correlation of miRNA and their target mRNA during three muscle development stages.

mRNA	miRNA	*R* value	mRNA	miRNA	*R* value
MYOD1	novel_6_20992	−0.5390881	MTSS1	novel_9_27936	−0.5860598
HBEGF	novel_6_20992	−0.7102848	SMARCD3	novel_10_2856	−0.5633121
ACHE	novel_X_29788	−0.7475892	RHOQ	novel_10_2856	−0.8857205
SRF	novel_X_29788	−0.9981668	CAPZA2	novel_4_17324	−0.9810114
CALR	novel_15_11149	−0.7613538	LIMK1	novel_9_27762	−0.9571368
GYLTL1B	novel_15_11149	−0.8369125	FHOD1	novel_9_27796	−0.6414053
METTL21A	novel_15_11149	−0.8442253	MEF2A	novel_6_22335	−0.8056134
WASL	novel_15_11149	−0.8928107	MTM1	novel_2_14454	−0.6334166
WASF3	novel_15_11149	−0.8967293	MTM1	novel_2_15515	−0.9702249
RASA1	novel_15_11149	−0.9028681	GPHN	novel_15_11290	−0.7468672
CSRP2	novel_15_11149	−0.9587139	LBX1	novel_16_11622	−0.9452654
EZR	novel_15_11149	−0.9827896	MEF2D	novel_13_6599	−0.9327755
PPP3CB	novel_4_18615	−0.6112719	IGFBP5	novel_4_18039	−0.9461923
MBNL1	novel_4_18615	−0.7985375	PDGFA	novel_13_7087	−0.9090636
FHOD1	novel_12_5322	−0.6604956	PDGFA	novel_5_19528	−0.8911486
ACADM	novel_12_5365	−0.9987412	AMOT	novel_3_15819	−0.577509
MET	novel_GL896243.1_30542	−0.6683036	SPTAN1	novel_2_15576	−0.8980043
SOD1	novel_GL896243.1_30542	−0.9586679	MTSS1	novel_12_5119	−0.7397887
RCAN1	novel_12_5576	−0.9999364	PDPK1	novel_17_12538	−0.9859897
SGCA	novel_1_1367	−0.9808128	WIPF1	novel_14_8940	−0.8267719
SRF	novel_1_1367	−0.8138354	CDC42BPB	novel_17_12654	−0.9731097
SIRT2	novel_X_29783	−0.8866714	SIX4	ssc-miR-744	−0.8646932
RXRG	ssc-miR-296-3p	−0.9848669	PROX1	ssc-miR-338	−0.9395592
NOS1	ssc-miR-296-3p	−0.963125	QKI	ssc-miR-423-3p	−0.9453357
MYL1	ssc-miR-497	−0.7053538	ACTN4	ssc-miR-133a-3p	−0.6850723
ACTA1	ssc-miR-1307	−0.7243173			

### Dual Luciferase Reporter Assay Validated the Interaction Between miRNAs and Their Target Genes

**Figure 6:**
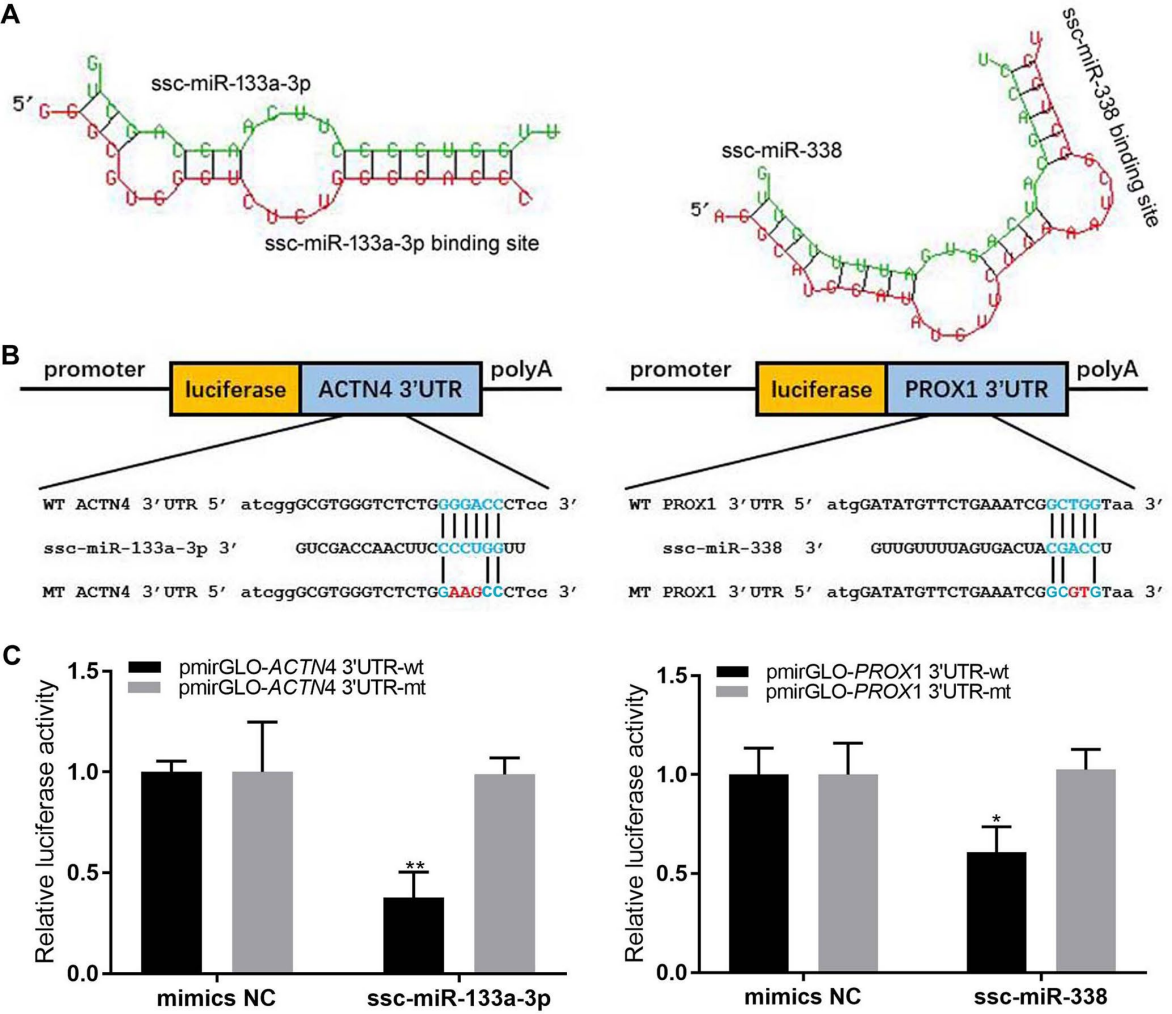
Dual luciferase reporter assay. **(A)** The secondary structure for miRNA and target gene 3′UTR. **(B)** Schematic of the wild-type and mutation-type 3′UTR vector with miRNA binding sites. Red is the mutation sites, and blue is the wild-type binding sites. **(C)** luciferase assays were performed.

We randomly selected two miRNA–mRNA pairs (*ACTN4*/ssc-miR-133a-3p *and Prox1*/ssc-miR-338), which showed significant negative expression correlations (*r* < −0.5) through all three skeletal muscle developmental stages, to verify whether the interaction between them were really exit. The dual luciferase reporter assay successfully validated the interaction between miRNA and their target genes. As shown in **Figure 6**, by binding to the 3′UTR region, all of the two miRNAs could markedly decrease the luciferase activity of the wild-type target genes (3′UTR-wt), while for the mutant type (3′UTR-mt), this repression was relieved. These results further confirmed the interaction between miRNA–mRNA pairs, which we discovered in the skeletal muscle development.

## Discussion

Transcriptome profiling is a good way to understand physiological functions and developmental regulations of organ tissues in plants and animals (Mcloughlin et al., 2014; Santos et al., 2014). The pig is not only an important livestock animal but also an important model organism in biomedical research. Therefore, a comprehensive atlas of gene expression for *S. scrofa* tissues and developmental stages is essential for both breeding and biomedical research (Yang et al., 2016). Here, we used RNA-seq to profile mRNAs and miRNAs found in nine different organ tissues and in three developmental stages of the Guizhou miniature pig, a breed widely used as a model organism in biomedical research. In all, the 18,576 PCGs we detected represent 85.97% of the annotated PCGs in the porcine reference genome. The numbers of PCGs ranged from 10,845 to 15,552 in different tissues and developmental stages, respectively, but only 46.14% of PCGs were constitutively expressed in all nine tissues. This indicates that PCG expressions are greatly temporally and spatially specific. Interestingly, the reproductive organs (ovary and testis) harbored the most complex transcriptomes, a finding consistent with those in humans and rats in which the testis and ovary also expressed the most genes (Yu et al., 2014).

We further analyzed the tissue-associated, tissue-specific, and universally expressed mRNA and miRNA across different tissues and showed that tissue-specific and -associated mRNAs and miRNAs are needed to maintain specific functions in a given tissue type. In this study, the numbers of tissue-specific and tissue-associated PCGs ranged from 15 to 992 and from 287 to 5,606, respectively. High variances reflected the differences in cell homogeneity and activity between different tissues. Tissues with higher transcriptional activities, such as testis and ovary, contained more associated and specific genes than other tissue types. The complex transcript of the Guizhou miniature pig testis was similar to that of other pig varieties. For instance, during the testis development of the Shaziling pig (a Chinese indigenous breed), 8,343 DEGs were identified and more than 50,000 miRNA–mRNA interaction sites were predicted (Ran et al., 2015). Additionally, many of these tissue-associated and tissue-specific genes are well correlated with physiological functions of each organ. For example, the testis-associated gene *PAK2* (p21-activated kinase 2) was reported to play a crucial regulatory role in porcine spermatogenesis apoptosis and when the *PAK2* gene was knocked down by related siRNA, the mitotic activity for Sertoli cells was significantly repressed (Ran et al., 2018b). Interestingly, while *PAK2* is one target gene of miR-26a, another miR-26a target gene, *ULK2*, was also a testis-associated gene in our study. *ULK2*, when knocked down, will inhibit swine Sertoli cell autophagy (Ran et al., 2018a). Meanwhile the testis-specific genes identified in this study such as *SPEM1*, *TNP1*, *PRM1*, *DAZL* (Hashemi et al., 2018), and *CABYR* (Shen et al., 2019) were also published as specific expression in human or mouse. As we know, tissue-specific functions are a result of specific expression and regulation of genes across an organism’s lifespan. The transcriptome’s degree of correlation suggests both similar and different biological functions between tissues. These data aid in the understanding of organ physiologies and molecular functions of genes in mammals.

Skeletal muscle is an important organ for maintaining movement and energy metabolism in animals (Liu et al., 2018), and pig skeletal muscle is a protein resource for humans (Tang et al., 2017; Yang et al., 2017). Thus, a systematic study of skeletal muscle development is essential to improving animal breeding as well as aiding biomedical research. Many studies have suggested that the PCGs, miRNAs, and the interactions between them are most important for cellular regulatory processes (Hou et al., 2016). Nielsen et al. (2010) analyzed the miRNA in pig longissimus dorsi by using deep sequencing, and they found that highly expressed miRNAs were involved in skeletal muscle development and regeneration (Nielsen et al., 2010). However, the understanding of skeletal muscle development based on a comprehensive profiling of mRNAs and miRNAs had been largely unclear. To fill that void, we carried out RNA-seq and small RNA-seq analysis on pig skeletal muscle at 0, 30, and 240 days after birth. In the D0 versus D30 and D30 versus D240 groups, 1,515 and 1,011 mRNAs, respectively, were differentially expressed. Functional analysis suggested the presence of significant differences in physiological characteristics at different developmental stages. Between D0 and D30, for example, genes functionally associated with translation, ribonucleoprotein complex biogenesis, and RNA and ncRNA processing were down-regulated, and in the D30 versus D240 groups, down-regulating genes were obviously involved in cell and biological adhesion and in skeletal system development.In the D0 versus D30 and D30 versus D240 groups, we detected 70 and 85 differentially expressed miRNAs, respectively. The miRNAs regulated biological processes by binding mRNAs at the 3′UTR. In the current study, 2194 negatively correlated (*r* < −0.5) miRNA–mRNA interaction pairs with binding sites for miRNAs at mRNA 3′UTRs were predicted. Of those pairs, 37 new miRNA–mRNA interaction pairs were associated with muscle cell differentiation and muscle organ development and were negatively correlated (*r* < −0.5) in the D0, D30, and D240 groups. Most of the predicted target mRNA in these pairs were reported to function in muscle. For instance, *SRF* and *MBNL1* (serum response factor and muscleblind-like splicing regulator 1) genes are reported to regulate muscle atrophy in mice (Collard et al., 2014), *AMOT* (the angiomotin gene) may influence human aortic smooth muscle cell migration (Wang et al., 2018a), and *QKI* (the protein quaking gene) regulates smooth muscle cell differentiation (Wu et al., 2017). Notable miRNAs that we found include ssc-miR-744, which is reported to significantly up-regulate in muscles after ischemia–reperfusion injury (Yang et al., 2015); ssc-miR-195, which induces postnatal quiescence of skeletal muscle stem cells (Sato et al., 2014); and ssc-miR-423-3p and ssc-miR-133a-3p, which each showed high correlations with mouse skeletal muscle C2C12 myoblast differentiation (Siengdee et al., 2015; Wang et al., 2018c). By targeting PCGs, miRNAs play important roles in regulating the complex processes of muscle development. Analysis of miRNA and mRNA expression profiles together was an effective way to minimize false-positive rates in miRNA–mRNA interaction pair predictions. In order to discover more miRNA–mRNA interaction pairs, we first analyzed the transcriptomes of both miRNA and mRNA in nine different tissues and then validated the miRNA–mRNA interaction pairs associated with muscle development in three different muscle development stages. For further verification, we also randomly selected two miRNA–mRNA interaction pairs to verify the expression profiles of each miRNA and its target mRNA by using a dual luciferase reporter assay. The results showed that, by binding to the 3′UTR region, miRNA could markedly decrease the luciferase activity of the target genes. Hence, the miRNA–mRNA interaction pairs predicted in this study most likely participate in the regulation of muscle development. The genes in these two pairs were *ACTN4*, a transcriptional regulator of myocyte enhancer factor that is associated with skeletal muscle differentiation (An et al., 2014), and *Prox1*, an essential gene for satellite cell differentiation and muscle fiber-type regulation (Kivela et al., 2016). They were paired, respectively, with important muscle-associated miRNAs ssc-miR-133a-3p (Wang et al., 2018c) and ssc-miR-338 (Mcdaneld et al., 2009). Although most of these miRNA–mRNA pairs have been reported to participate in muscle development, the actual interactions between them were still unclear and thus in need of further validation. The data from our study provide a rich resource for determining key interactions of miRNA–mRNA in muscle development.

## Data Availability

Sequencing data for miRNA have been deposited to Sequence Read Archive at the National Center for Biotechnical Information under accession number PRJNA552780. The RNA-seq data for mRNA were deposited in the Gene Expression Omnibus under accession number GSE73763.

## Ethics Statement

All animal handlings were approved by the Ethical Committee of the Faculty of Veterinary Medicine (EC2013/118) of Ghent University. All methods were performed in accordance with the relevant guidelines and regulations.

## Author Contributions

ZT and KL designed the experiment and wrote and revised the manuscript. MC and YaY wrote the manuscript and analyzed the data. YiY completed dual luciferase reporter assay. YT and SL were involved in sample collection and total RNA extraction. MZ installed the software used in this study.

## Funding

This work was supported by the National Natural Science Foundation of China (31830090), the National Key Project (2016ZX08009-003-006), the Shenzhen Science, Technology and Innovation Commission (JCYJ20170307160516413), the Special Fund for Industrial Development of Dapeng New Area at Shenzhen (KY20180114), and the Agricultural Science and Technology Innovation Program (ASTIP-AGIS5). The funders had no role in study design, data collection and analysis, decision to publish, or preparation of the manuscript.

## Conflict of Interest Statement

The authors declare that the research was conducted in the absence of any commercial or financial relationships that could be construed as a potential conflict of interest.

## References

[B1] An H. T. Kim J. Yoo S. Ko J. (2014). Small leucine zipper protein (sLZIP) negatively regulates skeletal muscle differentiation *via* interaction with alpha-actinin-4. J. Biol. Chem. 289, 4969–4979. 10.1074/jbc.M113.515395 24375477 PMC3931057

[B2] Anders S. Pyl P. T. Huber W. (2015). HTSeq—A Python framework to work with high-throughput sequencing data. Bioinformatics 31, 166–169. 10.1093/bioinformatics/btu638 25260700 PMC4287950

[B3] Arora A. Dey C. S. (2014). SIRT2 negatively regulates insulin resistance in C2C12 skeletal muscle cells. Biochim. Biophys. Acta 1842, 1372–1378. 10.1016/j.bbadis.2014.04.027 24793418

[B4] Bachmann C. Jungbluth H. Muntoni F. Manzur A. Y. Zorzato F. Treves S. (2017). Cellular, biochemical and molecular changes in muscles from patients with X-linked myotubular myopathy due to MTM1 mutations. Hum. Mol. Genet. 26, 320–332. 10.1093/hmg/ddw388 28007904

[B5] Bai L. Liang R. Yang Y. Hou X. Wang Z. Zhu S. (2015). MicroRNA-21 regulates PI3K/Akt/mTOR signaling by targeting TGFbetaI during skeletal muscle development in pigs. PLoS One 10, e0119396. 10.1371/journal.pone.0119396 25950587 PMC4423774

[B6] Blum R. Vethantham V. Bowman C. Rudnicki M. Dynlacht B. D. (2012). Genome-wide identification of enhancers in skeletal muscle: the role of MyoD1. Genes Dev. 26, 2763–2779. 10.1101/gad.200113.112 23249738 PMC3533080

[B7] Burguiere A. C. Nord H. Von Hofsten J. (2011). Alkali-like myosin light chain-1 (myl1) is an early marker for differentiating fast muscle cells in zebrafish. Dev. Dyn. 240, 1856–1863. 10.1002/dvdy.22677 21674687

[B8] Chakroun I. Yang D. Girgis J. Gunasekharan A. Phenix H. Kaern M. (2015). Genome-wide association between Six4, MyoD, and the histone demethylase Utx during myogenesis. FASEB J. 29, 4738–4755. 10.1096/fj.15-277053 26229056

[B9] Chao Z. Wu J. Zheng R. Li F. E. Xiong Y. Z. Deng C. Y. (2011). Molecular characterization and expression patterns of Lbx1 in porcine skeletal muscle. Mol. Biol. Rep. 38, 3983–3991. 10.1007/s11033-010-0516-1 21107715

[B10] Chen G. Masuda A. Konishi H. Ohkawara B. Ito M. Kinoshita M. (2016). Phenylbutazone induces expression of MBNL1 and suppresses formation of MBNL1-CUG RNA foci in a mouse model of myotonic dystrophy. Sci. Rep. 6, 25317. 10.1038/srep25317 27126921 PMC4850456

[B11] Collard L. Herledan G. Pincini A. Guerci A. Randrianarison-Huetz V. Sotiropoulos A. (2014). Nuclear actin and myocardin-related transcription factors control disuse muscle atrophy through regulation of Srf activity. J. Cell. Sci. 127, 5157–5163. 10.1242/jcs.155911 25344251

[B12] Dabaja A. A. Mielnik A. Robinson B. D. Wosnitzer M. S. Schlegel P. N. Paduch D. A. (2015). Possible germ cell-Sertoli cell interactions are critical for establishing appropriate expression levels for the Sertoli cell-specific microRNA, miR-202-5p, in human testis. Basic Clin. Androl. 25, 2. 10.1186/s12610-015-0018-z 25780590 PMC4349757

[B13] Ding X. Zhou S. Li M. Cao C. Wu P. Sun L. (2017). Upregulation of SRF is associated with hypoxic pulmonary hypertension by promoting viability of smooth muscle cells *via* increasing expression of Bcl-2. J. Cell. Biochem. 118, 2731–2738. 10.1002/jcb.25922 28176371

[B14] Dunaeva M. Blom J. Thurlings R. Pruijn G. J. M. (2018). Circulating serum miR-223-3p and miR-16-5p as possible biomarkers of early rheumatoid arthritis. Clin. Exp. Immunol. 193, 376–385. 10.1111/cei.13156 29892977 PMC6149957

[B15] Emrani R. Rebillard A. Lefeuvre L. Gratas-Delamarche A. Davies K. J. Cillard J. (2015). The calcineurin antagonist RCAN1-4 is induced by exhaustive exercise in rat skeletal muscle. Free Radic. Biol. Med. 87, 290–299. 10.1016/j.freeradbiomed.2015.06.023 26122706

[B16] Fortes M. A. Marzuca-Nassr G. N. Vitzel K. F. Da Justa Pinheiro C. H. Newsholme P. Curi R. (2016). Housekeeping proteins: how useful are they in skeletal muscle diabetes studies and muscle hypertrophy models? Anal. Biochem. 504, 38–40. 10.1016/j.ab.2016.03.023 27060530

[B17] Fougerousse F. Durand M. Suel L. Pourquie O. Delezoide A. L. Romero N. B. (1998). Expression of genes (CAPN3, SGCA, SGCB, and TTN) involved in progressive muscular dystrophies during early human development. Genomics 48, 145–156. 10.1006/geno.1997.5160 9521867

[B18] Friedlander M. R. Mackowiak S. D. Li N. Chen W. Rajewsky N. (2012). miRDeep2 accurately identifies known and hundreds of novel microRNA genes in seven animal clades. Nucleic Acids Res. 40, 37–52. 10.1093/nar/gkr688 21911355 PMC3245920

[B19] Giuffra E. Kijas J. M. Amarger V. Carlborg O. Jeon J. T. Andersson L. (2000). The origin of the domestic pig: independent domestication and subsequent introgression. Genetics 154, 1785–1791. 10747069 10.1093/genetics/154.4.1785 PMC1461048

[B20] Groenen M. A. Archibald A. L. Uenishi H. Tuggle C. K. Takeuchi Y. Rothschild M. F. (2012). Analyses of pig genomes provide insight into porcine demography and evolution. Nature 491, 393–398. 10.1038/nature11622 23151582 PMC3566564

[B21] Hashemi M. S. Mozdarani H. Ghaedi K. Nasr-Esfahani M. H. (2018). Among seven testis-specific molecular markers, SPEM1 appears to have a significant clinical value for prediction of sperm retrieval in azoospermic men. Andrology 6, 890–895. 10.1111/andr.12528 30054974

[B22] Herrmann J. Borkham-Kamphorst E. Haas U. Van De Leur E. Fraga M. F. Esteller M. (2006). The expression of CSRP2 encoding the LIM domain protein CRP2 is mediated by TGF-beta in smooth muscle and hepatic stellate cells. Biochem. Biophys. Res. Commun. 345, 1526–1535. 10.1016/j.bbrc.2006.05.076 16735029

[B23] Hou X. Tang Z. Liu H. Wang N. Ju H. Li K. (2012). Discovery of microRNAs associated with myogenesis by deep sequencing of serial developmental skeletal muscles in pigs. PLoS One 7, e52123. 10.1371/journal.pone.0052123 23284895 PMC3528764

[B24] Hou X. Yang Y. Zhu S. Hua C. Zhou R. Mu Y. (2016). Comparison of skeletal muscle miRNA and mRNA profiles among three pig breeds. Mol. Genet. Genomics 291, 559–573. 10.1007/s00438-015-1126-3 26458558

[B25] Hu Q. Tong H. Zhao D. Cao Y. Zhang W. Chang S. (2015). Generation of an efficient artificial promoter of bovine skeletal muscle alpha-actin gene (ACTA1) through addition of cis-acting element. Cell. Mol. Biol. Lett. 20, 160–176. 10.1515/cmble-2015-0009 26204400

[B26] Huang Da W. Sherman B. T. Lempicki R. A. (2009). Systematic and integrative analysis of large gene lists using DAVID bioinformatics resources. Nat. Protoc. 4, 44–57. 10.1038/nprot.2008.211 19131956

[B27] Huang D. W. Sherman B. T. Tan Q. Kir J. Liu D. Bryant D. (2007). DAVID Bioinformatics Resources: expanded annotation database and novel algorithms to better extract biology from large gene lists. Nucleic Acids Res. 35, W169–W175. 10.1093/nar/gkm415 17576678 PMC1933169

[B28] Iwakiri J. Terai G. Hamada M. (2017). Computational prediction of lncRNA–mRNA interactionsby integrating tissue specificity in human transcriptome. Biol. Direct 12, 15. 10.1186/s13062-017-0183-4 28595592 PMC5465533

[B29] Kaminski M. J. Kaminska M. Skorupa I. Kazimierczyk R. Musial W. J. Kaminski K. A. (2013). In-silico identification of cardiovascular disease-related SNPs affecting predicted microRNA target sites. Pol. Arch. Med. Wewn. 123, 355–363. 10.20452/pamw.1819 23648690

[B30] Kim D. Pertea G. Trapnell C. Pimentel H. Kelley R. Salzberg S. L. (2013). TopHat2: accurate alignment of transcriptomes in the presence of insertions, deletions and gene fusions. Genome Biol. 14, R36. 10.1186/gb-2013-14-4-r36 23618408 PMC4053844

[B31] Kim P. Park A. Han G. Sun H. Jia P. Zhao Z. (2018). TissGDB: tissue-specific gene database in cancer. Nucleic Acids Res. 46, D1031–D1038. 10.1093/nar/gkx850 29036590 PMC5753286

[B32] Kivela R. Salmela I. Nguyen Y. H. Petrova T. V. Koistinen H. A. Wiener Z. (2016). The transcription factor Prox1 is essential for satellite cell differentiation and muscle fibre-type regulation. Nat. Commun. 7, 13124. 10.1038/ncomms13124 27731315 PMC5064023

[B33] Koh W. Pan W. Gawad C. Fan H. C. Kerchner G. A. Wyss-Coray T. (2014). Noninvasive *in vivo* monitoring of tissue-specific global gene expression in humans. Proc. Natl. Acad. Sci. U. S. A. 111, 7361–7366. 10.1073/pnas.1405528111 24799715 PMC4034220

[B34] Kozomara A. Griffiths-Jones S. (2014). miRBase: annotating high confidence microRNAs using deep sequencing data. Nucleic Acids Res. 42, D68–D73. 10.1093/nar/gkt1181 24275495 PMC3965103

[B35] Kruger J. Rehmsmeier M. (2006). RNAhybrid: microRNA target prediction easy, fast and flexible. Nucleic Acids Res. 34, W451–W454. 10.1093/nar/gkl243 16845047 PMC1538877

[B36] Lage K. Hansen N. T. Karlberg E. O. Eklund A. C. Roque F. S. Donahoe P. K. (2008). A large-scale analysis of tissue-specific pathology and gene expression of human disease genes and complexes. Proc. Natl. Acad. Sci. U. S. A. 105, 20870–20875. 10.1073/pnas.0810772105 19104045 PMC2606902

[B37] Li B. Qing T. Zhu J. Wen Z. Yu Y. Fukumura R. (2017). A comprehensive mouse transcriptomic bodymap across 17 tissues by RNA-seq. Sci. Rep. 7, 4200. 10.1038/s41598-017-04520-z 28646208 PMC5482823

[B38] Li J. J. Huang H. Bickel P. J. Brenner S. E. (2014). Comparison of *D. melanogaster* and *C. elegans* developmental stages, tissues, and cells by modENCODE RNA-seq data. Genome Res. 24, 1086–1101. 10.1101/gr.170100.113 24985912 PMC4079965

[B39] Liang G. Yang Y. Niu G. Tang Z. Li K. (2017). Genome-wide profiling of *Sus scrofa* circular RNAs across nine organs and three developmental stages. DNA Res. 24, 523–535. 10.1093/dnares/dsx022 28575165 PMC5737845

[B40] Lin H. Y. Cheng C. H. Chen D. T. Chen Y. A. Park J. Y. (2016). Coexpression and expression quantitative trait loci analyses of the angiogenesis gene–gene interaction network in prostate cancer. Transl. Cancer Res. 5, S951–S963. 10.21037/tcr.2016.10.55 28664150 PMC5485921

[B41] Liu H. Xi Y. Liu G. Zhao Y. Li J. Lei M. (2018). Comparative transcriptomic analysis of skeletal muscle tissue during prenatal stages in Tongcheng and Yorkshire pig using RNA-seq. Funct. Integr. Genomics 18, 195–209. 10.1007/s10142-017-0584-6 29322263

[B42] Love M. I. Huber W. Anders S. (2014). Moderated estimation of fold change and dispersion for RNA-seq data with DESeq2. Genome Biol. 15, 550. 10.1186/s13059-014-0550-8 25516281 PMC4302049

[B43] Mcdaneld T. G. Smith T. P. Doumit M. E. Miles J. R. Coutinho L. L. Sonstegard T. S. (2009). MicroRNA transcriptome profiles during swine skeletal muscle development. BMC Genomics 10, 77. 10.1186/1471-2164-10-77 19208255 PMC2646747

[B44] Mcloughlin K. E. Nalpas N. C. Rue-Albrecht K. Browne J. A. Magee D. A. Killick K. E. (2014). RNA-seq transcriptional profiling of peripheral blood leukocytes from cattle infected with *Mycobacterium bovis* . Front. Immunol. 5, 396. 10.3389/fimmu.2014.00396 25206354 PMC4143615

[B45] Mora A. Davies A. M. Bertrand L. Sharif I. Budas G. R. Jovanovic S. (2003). Deficiency of PDK1 in cardiac muscle results in heart failure and increased sensitivity to hypoxia. EMBO J. 22, 4666–4676. 10.1093/emboj/cdg469 12970179 PMC212735

[B46] Nawrocki E. P. Burge S. W. Bateman A. Daub J. Eberhardt R. Y. Eddy S. R. (2015). Rfam 12.0: updates to the RNA families database. Nucleic Acids Res. 43, D130–D137. 10.1093/nar/gku1063 25392425 PMC4383904

[B47] Neville M. J. Collins J. M. Gloyn A. L. Mccarthy M. I. Karpe F. (2011). Comprehensive human adipose tissue mRNA and microRNA endogenous control selection for quantitative real-time-PCR normalization. Obesity (Silver Spring) 19, 888–892. 10.1038/oby.2010.257 20948521 PMC4623139

[B48] Nielsen M. Hansen J. H. Hedegaard J. Nielsen R. O. Panitz F. Bendixen C. (2010). MicroRNA identity and abundance in porcine skeletal muscles determined by deep sequencing. Anim. Genet. 41, 159–168. 10.1111/j.1365-2052.2009.01981.x 19917043

[B49] Park M. Lee B. S. Jeon S. H. Nam H. J. Lee G. Kim C. H. (2015). A novel isoform of met receptor tyrosine kinase blocks hepatocyte growth factor/Met signaling and stimulates skeletal muscle cell differentiation. J. Biol. Chem. 290, 1804–1817. 10.1074/jbc.M114.596957 25471370 PMC4340422

[B50] Peng Y. Xiang H. Chen C. Zheng R. Chai J. Peng J. (2013). MiR-224 impairs adipocyte early differentiation and regulates fatty acid metabolism. Int. J. Biochem. Cell. Biol. 45, 1585–1593. 10.1016/j.biocel.2013.04.029 23665235

[B51] Ran M. Chen B. Wu M. Liu X. He C. Yang A. (2015). Integrated analysis of miRNA and mRNA expression profiles in development of porcine testes. RSC Adv. 5, 63439–63449. 10.1039/C5RA07488F

[B52] Ran M. Li Z. Cao R. Weng B. Peng F. He C. (2018a). miR-26a suppresses autophagy in swine Sertoli cells by targeting ULK2. Reprod. Domest. Anim. 53, 864–871. 10.1111/rda.13177 29761550

[B53] Ran M. Weng B. Cao R. Li Z. Peng F. Luo H. (2018b). miR-26a inhibits proliferation and promotes apoptosis in porcine immature Sertoli cells by targeting the PAK2 gene. Reprod. Domest. Anim. 53, 1375–1385. 10.1111/rda.13254 30024056

[B54] Riffo-Campos A. L. Riquelme I. Brebi-Mieville P. (2016). Tools for sequence-based miRNA target prediction: what to choose? Int. J. Mol. Sci. 17, E1987. 10.3390/ijms17121987 27941681 PMC5187787

[B55] Roux J. Gonzalez-Porta M. Robinson-Rechavi M. (2012). Comparative analysis of human and mouse expression data illuminates tissue-specific evolutionary patterns of miRNAs. Nucleic Acids Res. 40, 5890–5900. 10.1093/nar/gks279 22457063 PMC3401464

[B56] Runfola V. Sebastian S. Dilworth F. J. Gabellini D. (2015). Rbfox proteins regulate tissue-specific alternative splicing of Mef2D required for muscle differentiation. J. Cell. Sci. 128, 631–637. 10.1242/jcs.161059 25609712 PMC4357529

[B57] Sakellariou G. K. Mcdonagh B. Porter H. Giakoumaki I. I. Earl K. E. (2018). Comparison of whole body SOD1 knockout with muscle-specific SOD1 knockout mice reveals a role for nerve redox signaling in regulation of degenerative pathways in skeletal muscle. Antioxid. Redox Signal. 28, 275–295. 10.1089/ars.2017.7249 29065712 PMC5743036

[B58] Santos C. A. Blanck D. V. De Freitas P. D. (2014). RNA-seq as a powerful tool for penaeid shrimp genetic progress. Front. Genet. 5, 298. 10.3389/fgene.2014.00298 25221571 PMC4147233

[B59] Sato T. Yamamoto T. Sehara-Fujisawa A. (2014). miR-195/497 induce postnatal quiescence of skeletal muscle stem cells. Nat. Commun. 5, 4597. 10.1038/ncomms5597 25119651

[B60] Shen S. Li D. Liang J. Wang J. (2019). Testis-specific calcium-binding protein CBP86-IV (CABYR) binds with phosphoglycerate kinase 2 *in vitro* and *in vivo* experiment. Andrologia 10, e13287. 10.1111/and.13287 30972801

[B61] Siengdee P. Trakooljul N. Murani E. Schwerin M. Wimmers K. Ponsuksili S. (2015). MicroRNAs regulate cellular ATP levels by targeting mitochondrial energy metabolism genes during C2C12 myoblast differentiation. PLoS One 10, e0127850. 10.1371/journal.pone.0127850 26010876 PMC4444189

[B62] Sromek M. Glogowski M. Chechlinska M. Kulinczak M. Szafron L. Zakrzewska K. (2017). Changes in plasma miR-9, miR-16, miR-205 and miR-486 levels after non-small cell lung cancer resection. Cell. Oncol. (Dordr) 40, 529–536. 10.1007/s13402-017-0334-8 28634901 PMC13001572

[B63] Staus D. P. Blaker A. L. Medlin M. D. Taylor J. M. Mack C. P. (2011). Formin homology domain-containing protein 1 regulates smooth muscle cell phenotype. Arterioscler. Thromb. Vasc. Biol. 31, 360–367. 10.1161/ATVBAHA.110.212993 21106951 PMC3025477

[B64] Szabo L. Morey R. Palpant N. J. Wang P. L. Afari N. Jiang C. (2015). Statistically based splicing detection reveals neural enrichment and tissue-specific induction of circular RNA during human fetal development. Genome Biol. 16, 126. 10.1186/s13059-015-0690-5 26076956 PMC4506483

[B65] Tallquist M. D. Weismann K. E. Hellstrom M. Soriano P. (2000). Early myotome specification regulates PDGFA expression and axial skeleton development. Development 127, 5059–5070. 11060232 10.1242/dev.127.23.5059

[B66] Tang Z. Wu Y. Yang Y. Yang Y. T. Wang Z. Yuan J. (2017). Comprehensive analysis of long non-coding RNAs highlights their spatio-temporal expression patterns and evolutional conservation in Sus scrofa. Sci. Rep. 7, 43166. 10.1038/srep43166 28233874 PMC5324117

[B67] Tang Z. Yang Y. Wang Z. Zhao S. Mu Y. Li K. (2015). Integrated analysis of miRNA and mRNA paired expression profiling of prenatal skeletal muscle development in three genotype pigs. Sci. Rep. 5, 15544. 10.1038/srep15544 26496978 PMC4620456

[B68] Villmow M. Klockner U. Heymes C. Gekle M. Rueckschloss U. (2015). NOS1 induces NADPH oxidases and impairs contraction kinetics in aged murine ventricular myocytes. Basic Res. Cardiol. 110, 506. 10.1007/s00395-015-0506-5 26173391

[B69] Wang H. Zhang P. Chen W. Feng D. Jia Y. Xie L. X. (2012a). Evidence for serum miR-15a and miR-16 levels as biomarkers that distinguish sepsis from systemic inflammatory response syndrome in human subjects. Clin. Chem. Lab. Med. 50, 1423–1428. 10.1515/cclm-2011-0826 22868808

[B70] Wang X. Du C. He X. Deng X. He Y. Zhou X. (2018a). MiR-4463 inhibits the migration of human aortic smooth muscle cells by AMOT. Biosci. Rep. 38, BSR20180150. 10.1042/BSR20180150 29752344 PMC6147913

[B71] Wang X. Zhang P. Li L. Che D. Li T. Li H. (2018b). miRNA editing landscape reveals miR-34c regulated spermatogenesis through structure and target change in pig and mouse. Biochem. Biophys. Res. Commun. 502, 486–492. 10.1016/j.bbrc.2018.05.197 29864426

[B72] Wang X. G. Yu J. F. Zhang Y. Gong D. Q. Gu Z. L. (2012b). Identification and characterization of microRNA from chicken adipose tissue and skeletal muscle. Poult. Sci. 91, 139–149. 10.3382/ps.2011-01656 22184439

[B73] Wang Y. Ma J. Qiu W. Zhang J. Feng S. Zhou X. (2018c). Guanidinoacetic acid regulates myogenic differentiation and muscle growth through miR-133a-3p and miR-1a-3p co-mediated Akt/mTOR/S6K signaling pathway. Int. J. Mol. Sci. 19, E2837. 10.3390/ijms19092837 30235878 PMC6163908

[B74] Wu Y. Li Z. Yang M. Dai B. Hu F. Yang F. (2017). MicroRNA-214 regulates smooth muscle cell differentiation from stem cells by targeting RNA-binding protein QKI. Oncotarget 8, 19866–19878. 10.18632/oncotarget.15189 28186995 PMC5386729

[B75] Yang J. C. Wu S. C. Rau C. S. Chen Y. C. Lu T. H. Wu Y. C. (2015). TLR4/NF-kappaB-responsive microRNAs and their potential target genes: a mouse model of skeletal muscle ischemia–reperfusion injury. Biomed. Res. Int. 2015, 410721. 10.1155/2015/410721 25692136 PMC4321099

[B76] Yang Y. Liang G. Niu G. Zhang Y. Zhou R. Wang Y. (2017). Comparative analysis of DNA methylome and transcriptome of skeletal muscle in lean-, obese-, and mini-type pigs. Sci. Rep. 7, 39883. 10.1038/srep39883 28045116 PMC5206674

[B77] Yang Y. Zhou R. Mu Y. Hou X. Tang Z. Li K. (2016). Genome-wide analysis of DNA methylation in obese, lean, and miniature pig breeds. Sci. Rep. 6, 30160. 10.1038/srep30160 27444743 PMC4957084

[B78] Yu Y. Fuscoe J. C. Zhao C. Guo C. Jia M. Qing T. (2014). A rat RNA-seq transcriptomic BodyMap across 11 organs and 4 developmental stages. Nat. Commun. 5, 3230. 10.1038/ncomms4230 24510058 PMC3926002

[B79] Yuan H. Niu Y. Liu X. Fu L. (2014). Exercise increases the binding of MEF2A to the Cpt1b promoter in mouse skeletal muscle. Acta Physiol. (Oxf) 212, 283–292. 10.1111/apha.12395 25213552

[B80] Zeng J. Liu S. Zhao Y. Tan X. Aljohi H. A. Liu W. (2016). Identification and analysis of house-keeping and tissue-specific genes based on RNA-seq data sets across 15 mouse tissues. Gene 576, 560–570. 10.1016/j.gene.2015.11.003 26551299

[B81] Zhang W. R. Zhang H. N. Wang Y. M. Dai Y. Liu X. F. Li X. (2017). miR-143 regulates proliferation and differentiation of bovine skeletal muscle satellite cells by targeting IGFBP5. In Vitro Cell. Dev. Biol. Anim. 53, 265–271. 10.1007/s11626-016-0109-y 27800570

[B82] Zhang X. Azhar G. Williams E. D. Rogers S. C. Wei J. Y. (2015a). MicroRNA clusters in the adult mouse heart: age-associated changes. Biomed. Res. Int. 2015, 732397. 10.1155/2015/732397 26221604 PMC4499379

[B83] Zhang Y. Jing J. Li X. Wang J. Feng X. Cao R. (2015b). Integration analysis of miRNA and mRNA expression profiles in swine testis cells infected with Japanese encephalitis virus. Infect. Genet. Evol. 32, 342–347. 10.1016/j.meegid.2015.03.037 25847692

